# Qualitative study on the perception of good death in patients with end-stage cancer in oncology nurses

**DOI:** 10.1186/s12912-024-02081-x

**Published:** 2024-06-25

**Authors:** Wei-dan Wu, Yi Wang, Xin-yu Fu, Jin-hua Zhang, Chen-yang Zhang, Xin-Li Mao, Shao-wei Li

**Affiliations:** 1 Department of Gastroenterology, Taizhou Hospital of Zhejiang Province affiliated to Wenzhou Medical University, Linhai, Zhejiang Province China; 2 Key Laboratory of Minimally Invasive Techniques & Rapid Rehabilitation of Digestive System Tumor of Zhejiang Province, Taizhou Hospital of Zhejiang Province affiliated to Wenzhou Medical University, Linhai, Zhejiang Province China; 3 Institute of Digestive Disease, Taizhou Hospital of Zhejiang Province Affiliated to Wenzhou Medical University, Linhai, Zhejiang Province China; 4 Taizhou Hospital of Zhejiang Province affiliated to Wenzhou Medical University, Linhai, Zhejiang Province China

**Keywords:** Oncology department, Nurse, Good death, Cognition, Qualitative research

## Abstract

**Objective:**

To explore the perception of good death of patients with end-stage cancer by nurses in the oncology department.

**Method:**

In the study we used a phenomenological approach and semi-structured interviews. A total of 11 nurses from the oncology department of a Grade A hospital in Taizhou were interviewed on the cognition of good death from July 1 to September 30, 2022. Colaizzi’s analysis method was used to analyse the interview data. This study followed the consolidated criteria for reporting qualitative research (COREQ).

**Result:**

Four themes were identified: a strong sense of responsibility and mission; To sustain hope and faith; The important role of family members; Improve patients’ quality of life.

**Conclusion:**

The nurses in the department of oncology have a low level of knowledge about the “good death”, and the correct understanding and view of the “good death” is the premise of the realization of " good death”. The ability of nursing staff to improve the “good death”, attention, and meet the needs and wishes of individuals and families, is the guarantee of the realization of “good death”.

**Supplementary Information:**

The online version contains supplementary material available at 10.1186/s12912-024-02081-x.

## Introduction

The 2020 Global Cancer Statistics, published by the American Cancer Society shows that there were 10 million cancer deaths worldwide in 2020, and the global cancer burden is projected to reach 28.4 million by 2040, a 47% increase over 2020 [1]. Our country leads the world in cancer incidence and mortality [1], and the health damage caused by cancer is almost twice the world average [2, 3].

Patients with end-stage cancer are defined as those who have no hope of a cure in modern medicine and are expected to survive for 3–6 months [4, 5]. The terminal cancer patient’s condition can not be reversed, and has not yet effectively alleviated the pain, and the use of life support measures such as breathing machines to some extent prolonged the pain, resulting in patients can not die in comfort and dignity [6]. Death represents a significant and inevitable stage in the cycle of life, marking its final chapter for all living beings. It is a profound and crucial period that holds tremendous significance in the grand scheme of existence [7]. Hospice care is a holistic approach aimed at addressing the physical, psychosocial, and spiritual needs of individuals with a terminal illness and their family members. It provides comprehensive support and services to ensure comfort, dignity, and quality of life during this challenging time [8]. There is substantial evidence demonstrating the positive impacts of hospice care. It has been shown to enhance the quality of end-of-life (EoL) care, reduce medical costs, align with individuals’ preferences for comfort-focused care, and minimize the use of burdensome therapies. These findings support the value and effectiveness of hospice care in providing appropriate and compassionate support to patients and their families during the terminal stages of illness [9, 10].

Hospice care in China is still in its early stages compared to certain Western countries. Efforts are being made to expand access to hospice services, raise awareness about the benefits of palliative care, and improve the quality of care provided to individuals with life-limiting illnesses in China [11]. As the concept of “eugenics” and “optimal parenting” gains popularity, the idea of a “good death” is gradually being brought to the forefront. It not only reflects the respect for life but also signifies the progress of society and civilization. The hospice concept was introduced in China in the 1980s. The concept and characteristics of a “good death” originated from early end-of-life care, with the ultimate goal of advocating for people’s support in the field of end-of-life care and drawing attention to the well-being of terminally ill patients. In 1998, Emanuel et al. proposed the framework for a good death, providing a comprehensive explanation of the multidimensional personal experience encompassed by death. Researchers divided the process of death into four key components: the inherent characteristics of the patient, the variable factors within the patient’s experience, the interventions by the healthcare system, and the outcomes. A “good death” can be described as one that occurs without the knowledge of the exact time of death, enables the individual to bid farewell to loved ones, avoids unnecessary interventions, allows the person to have some control over the place of death, minimizes distress and suffering, respects the patient’s and their family’s wishes, and aligns reasonably with clinical, cultural, and ethical standards. This comprehensive definition encompasses multiple aspects that contribute to a positive and meaningful end-of-life experience for both the individual and their loved ones [12, 13].

Across different cultures, certain attributes of a good death are often emphasized. These include maintaining a pain-free status through effective pain management, providing emotional comfort and support to the person and their loved ones, and ensuring that individuals are prepared for the inevitability of death through open communication and appropriate end-of-life planning. These attributes are recognized as important factors in promoting a more peaceful and dignified transition at the end of life, regardless of cultural backgrounds [14, 15]. Nurses who have a good understanding of the concept of a “good death” are better equipped to provide more effective end-of-life care to patients [16]. When providing care to individuals who are dying, nurses may experience a range of emotions, including anger, despair, distress, and guilt [17, 18]. Understanding the concept of a good death and accepting the inevitability of death can aid nurses in coping with these complex emotions. However, it is worth noting that the acceptance of hospice care in Chinese society has been relatively slow, despite its introduction to mainland China as early as 1988 [19]. With the aging population, there is an increasing demand for end-of-life care. To measure this demand, an index system can be used, taking into account factors such as the burden caused by diseases, the dependency ratio of the elderly population, and the speed of aging [20]. In China, the objective demand for end-of-life care is indeed increasing. However, traditional cultural influences often make discussions about death taboo, and the concept of a “good death” is not widely accepted by most people [19].

Good Death (GD) is one of the core objectives of hospice care [21, 22]. This study conducted in-depth interviews with nurses in the department of oncology to understand the current implementation of good death technology, the cognitive status of medical staff on good death, and the clinical coping strategies for patients with end-stage cancer, to determine the cognitive deficiencies of medical staff in good death and the aspects of continuous learning. The study mentioned focused on oncology inpatient unit nurses because they are frequently involved in providing end-of-life care. As patients with cancer often face end-of-life issues, it is important to understand the experiences and perspectives of nurses working in this specific setting.

## Methods

### Study design

We used phenomenological qualitative research and face-to-face semi-structured interviews to explore the perception of good death of patients with end-stage cancer by nurses in the oncology department in Taizhou Hospital of Zhejiang Province from July 1 to September 30, 2022. In qualitative research, phenomenological methods focus on describing common experiences shared by the entirepopulation, which also helps researchers to engage with participants from an in-depth perspective and to understand their experiences. Our research team has extensive experience in qualitative research.

### Participants and ethical considerations

Purposive sampling was employed to select the participants who were eligible and could provide rich information about the research question.

Inclusion criteria: (1) Nurses with a license to practice nursing; (2) Oncology nurses with a minimum of 6 months of clinical nursing experience; (3) Providing care services to terminally ill cancer patients, and have work experience in hospice; (4) Ability to clearly articulate their views; (5) Providing informed consent and voluntary participation in this study. Exclusion criteria: (1) Nurses who withdrew from the interview process; (2) Nurses who were on leave or engaged in training, resulting in an absence from their position for more than 3 months; (3) Nurses who were unwilling to discuss their experiences in caring for terminally ill cancer patients.

In this study, the report will replace each participant with a code, and the interviewee’s identity, contact information will not be disclosed to others. Sound content is also used only in this study. The study was reviewed and approved by the Ethics Committee of Taizhou Hospital, Zhejiang Province, China (approval number: K20220789).

The qualitative data collection method employed for this study involved semi-structured, face-to-face interviews. Prior to the start of each interview, all nurses were provided with written informed consent to participate in the research. All interviews were digitally recorded, assigned pseudonyms, and transcribed verbatim. We took measures to ensure that the participants understood the purpose and process of the study, and we emphasized the privacy of the interview environment and the confidentiality of the data. The interviews will take place within the confines of the hospital’s designated conversational chambers, ensuring utmost privacy for the participants. Saturation was considered to be reached when no new themes emerged from the inductive content analysis. In total, we conducted interviews with 11 members of the oncology nursing team (See Table 1).

**Table 1:** General information of research objects (*n*=11)

Number	Gender	Age	Years of working
1	female	33	9
2	female	36	12
3	female	42	17
4	female	26	1
5	female	28	5
6	female	29	4
7	female	39	17
8	female	37	14
9	female	32	8
10	female	30	6
11	female	25	3

### Data collection

During the interview, subjects were also given the opportunity to read the consent form, confirm understanding, and ask questions. Verbal consent was obtained to preserve the anonymity of the subjects. During the interviews, participants were offered explanations for any inquiries they had. Additionally, participants had the option to refuse further interviews and withdraw from the study for any reason. In addition, two oncology nurses were selected for a pre-interview prior to data collection to ensure the clarity of the questions and to identify any potential problems. The data from preliminary interviews was not included in this study but was utilized to modify the interview structure based on the preliminary findings. The preinterviews were treated as tests and were excluded from the analysis. The final interview used in this study included the items are listed in Table 2. Interviews were conducted in a quiet consultation room at the hospital between July 1 and September 30, 2022. Each person was interviewed one time, and each interview lasted approximately 30–50 min. All the interviews were conducted by a nurse with master who was trained in qualitative research. A research assistant played an auxiliary role which included recording the interviews.

**Table 2:** Interview outline

Interview guide	
1	What factors do you think are related to achieving a good death
2	What would you do to help terminal cancer patients achieve a good death
3	Your experience caring for terminal patients, your changed understanding of death
4	What do you think can be done to improve the quality of life of patients with end-stage cancer
5	Do you think your current techniques for achieving a good death are sufficient to help patients achieve a good death

The investigator audio recorded with permission, and participants’ responses, including nonverbal cues and body language during the interviews, were noted. The results will be returned to each participant within 24 h of each interview, to verify the interview details, thus ensuring the accuracy and credibility of the analysis. Before this interview, investigators were trained in interview and communication skills, including effective listening and giving positive feedback, establishing good relationships with interviewees, maintaining eye contact, not interrupting interviewees, not judging their views, etc.

### Data analysis

Audio recordings were transcribed verbatim and checked for accuracy by repeated listening within 24 h of the interviews. After the interview, the data were analysed separately and immediately by two researchers with skilled analysis experience. Interview data was analysed using Nvivo12.0, a computer-assisted qualitative data management software. Colaizzi’s phenomenological seven-stepmethod was used for data analysis to complete theextraction of themes and sub-themes regarding the perception of good death of patients with end-stage cancer among oncology nurses (see Table 3). Any disagreement between researchers was resolved by making decisions through discussion until a consensus was reached. The final transcribed data, as well as the extracted themesand sub-themes, were sent to the participants simultaneously, and all participants agreed to be contacted again. This study met the criteria of Consolidated Criteria for Reporting Qualitative Studies (COREQ).

**Table 3:** Colaizzi’s seven-step process for qualitative data analysis

NO	Data analysis step
1	All interviews were recorded and transcribed. Each transcript was carefully read several times.
2	Researchers re-read, highlighted, and extracted meaningful statements directly related to the perception of good death of patients with end-stage cancer by nurses in the oncology department.
3	Meanings from all significant statements were summarized.
4	Identified and organized the formulated meanings into theme clusters. The researchers compared the theme clusters with the original data several times to determine consistency.
5	Exhaustively described the investigated phenomenon of the perception of good death of patients with end-stage cancer by nurses in the oncology department.
6	Recognized similar subthemes, identified the basic structure, and obtained the main themes.
7	Returned to the participants to confirm the findings. The authors discussed their disagreements until a consensus was reached.

### Rigor and trustworthiness

To ensure the study’s dependability, the methods and analyses used were described in detail. The study interviewer was a master’s degree student in nursing. The interviewer received systematic qualitative training to master qualitative research methods, was experienced in oncology practices, and established a good relationship with the participants before the interviews commenced. This facilitated the acquisition of real information. The researcher maintained a neutral attitude during the interview, did not lead or hint, did not interrupt the interviewee at will, and only asked timely follow-up questions, rhetorical questions, and clarifications until no new information emerged. Therefore, credibility was ensured. The collection, analysis, and interpretation of data were continually reviewed and detailed to ensure its dependability. The data extracted from the survey results were described in detail to achieve confirmability. Regarding transferability, this study described in detail the inclusion criteria, exclusion criteria, and demographic characteristics involved. Simultaneously, the Consolidated Criteria for Reporting Qualitative Research (COREQ) checklist was used to report the findings (See Appendix I for details).

## Result

**Fig. 1:**
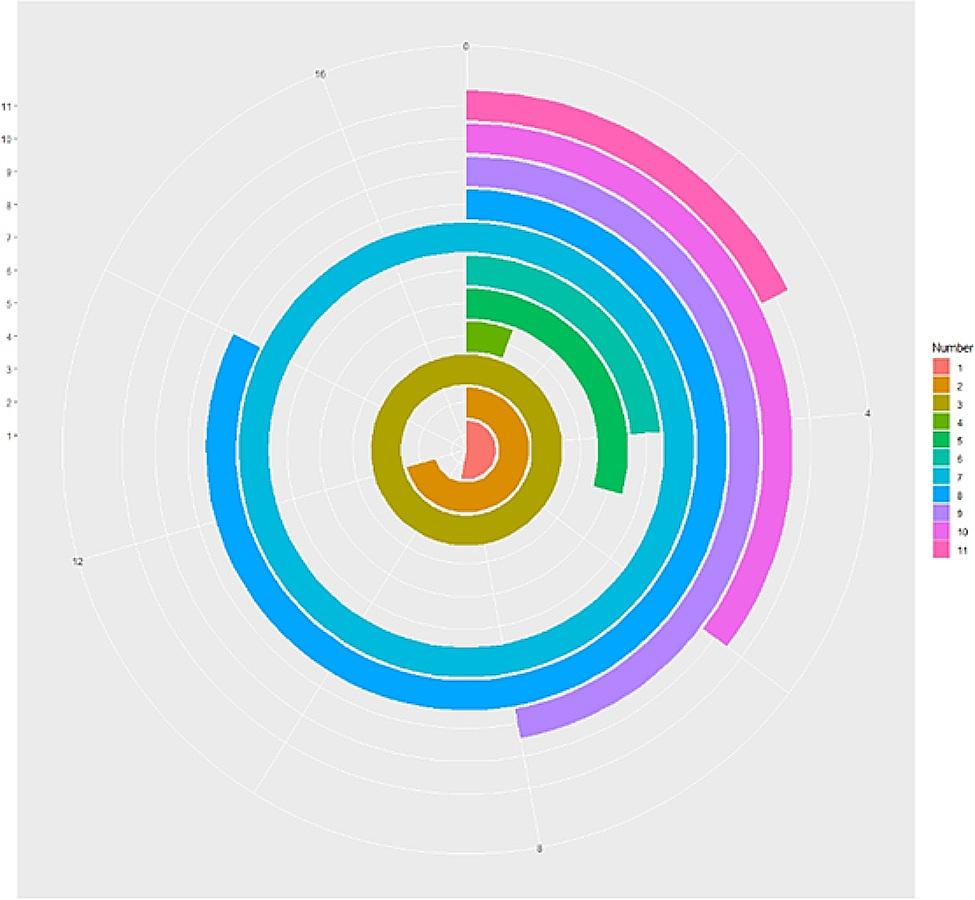
A visual analysis of the working years of the 11 participants included

Characteristics of the 11 participants are shown in Table 1. All eleven participants were female. The shortest term of employment as a Registered Nurse was one and a half years, and the longest was 17 years (Fig. 1). Four distinct themes emerged from analysis of the interview data: (1) A strong sense of responsibility and mission; (2) Sustaining hope and faith; (3) The important role of family members; and (4) Improving patients’ quality of life. Each theme included three–four subthemes (see Table 4).

**Table 4:** Themes and subthemes

Theme	Subtheme
A strong sense of responsibility and mission	
Sustaining hope and faith	Patient confidentiality Moral support
The important role of family members	The accompanying role of family members The communication role of family members The caregiving role of family members
Improving patients’ quality of life	Symptom control Palliative care

### Theme one: a strong sense of responsibility and mission

Most respondents said that when they realized that a patient was dying, their presence was more important than ever, triggering a strong sense of responsibility and mission. “End-stage patients will leave at any time, when in the dying patient evaluation period, I will often ward, observe the patient’s vital signs, keep the comfort of family members, at this time of the patients and family members are in great need of medical personnel to accompany and support, especially families, at this point the heart is very fragile, especially need a psychological support.” (A5, female, 28 y.o) “For the families of the patients whose death is imminent, I will tell them to tell the patients as soon as possible if there is anything they need to tell them. If there is anything that needs the help of our doctors and nurses, they can tell us at any time. We will do our best to help.” (A7, female, 39 y.o).

### Theme two: sustaining hope and faith

#### Patient confidentiality

Under the influence of traditional Chinese culture, when patients enter the terminal stage of cancer, considering the patients’ physical and psychological conditions and psychological acceptance of the disease, medical staff needs to inform the patients’ families and seek treatment advice, whether to conceal the true condition of patients [23], whether to continue treatment or give up treatment and so on so that the whole family is faced with a major choice. In this interview, the interviewees discussed their views on the confidentiality of the patient’s condition from the point of view of good death, and the nurses had better cognition of the confidentiality of the patient’s condition. “We have a lot of family members who are concerned about the patient’s ability to cope. They tell us in advance not to discuss the patient’s condition in front of the patient, and they ask us to keep it confidential when the patient asks. We usually comply with their request at this time. “(A9, female, 32 y.o).

“A lot of family members ask for the patient’s condition to be kept confidential. Our doctors and nurses usually communicate on how to settle accounts with patients in a unified way. “(A3, female, 42 y.o).

#### Moral support

Patients and family members experience a complex range of emotions after being informed of a cancer diagnosis, and how to make patients and family members accept the reality that cancer is incurable is a challenge for healthcare professionals [24–27]. In the interview, the interviewees mentioned the importance of spiritual support for cancer families. “Many patients have no faith. I have seen many patients who have been in a period of anger after learning that they have cancer. They think why they are so unlucky. They have this disease and have no interest in doing anything. They think that the world is unfair and cruel to them. If we can help them to seek their faith, such as religion, it may have some spiritual comfort for them. " (A8, female, 37 y.o) “I met a retired civil servant in my work who, after learning that he had cancer, organized his years of Work Records and compiled a memoir by year. I think he would review his experiences and values when he read these memoirs, and his heart would be at peace for a while. I got an idea from him that I could use a similar approach to help other patients and their families find value in their lives and live more peacefully in the final stages of their lives. " (A6, female, 29 y.o).

### Theme three: the important role of family members

#### The accompanying role of family members

Influenced by our traditional culture, most terminal cancer patients want their closest family members to be with them at the end of their lives. Most of the interviewees indicated that the accompany of family members is a comfort to the patients, which makes them feel that they are loved and meets their psychological needs [28]. “Patients at this stage are more psychologically vulnerable than those with other diseases. At the end of their lives, the company of their family members is a great psychological comfort to them. Although I can’t have a lot of company during an epidemic, I usually ask one of the family members to stay here to accompany the patient. “(A2, female, 36 y.o) “At this time, the family member will stay by the patient’s side. Even if they don’t do anything or say anything, the patient will feel that they are cared for by someone and feel that they are still loved. “(A10, female, 30 y.o).

#### The communication role of family members

Under the influence of the Chinese traditional concept of life and death, there are still some difficulties in implementing and promoting euthanasia, especially for cancer patients, whose families often choose to hide the true situation from them, medical staff can only discuss it with their families [29, 30]. The nurses mentioned that most of the family members lack the methods of psychological care and the experience of taking care of terminal cancer patients, do not know the psychological needs of the terminal cancer patients, and can not do the psychological work of the patients in time and effectively. “During the work process, some patients’ psychological needs are very high, but the family members accompanying them don’t understand the patients’ psychological needs. The two of them can’t chat together and have nothing to say for a whole day. “(A4, female, 26 y.o).

#### The caregiving role of family members

Family members accompany patients for a long time, know the daily living habits of patients best, can provide wholehearted care, and can timely detect and feedback on the symptoms of patients and changes in their condition, the care of family members for patients is an important component of the medical staff to evaluate the patient’s good death. “Many patients at this stage due to pain and other effects, the ability to move limited, and many daily activities need family care.” (A11, female, 25 y.o) “Terminally ill patients, ECG monitoring everyday detection of vital signs, we usually also one hour patrol, family members beside can pay attention to the patient’s vital signs changes. " (A1, female, 33 y.o).

### Theme four: improving patients’ quality of life

#### Symptom control

Most interviewees believed that end-stage patients should focus on symptom control and pain relief. “In the end, the most uncomfortable thing for many patients is the cancer pain, which makes them unable to move when they turn over. Taking painkillers and injecting painkillers can no longer control the pain. If it can reduce their pain, it is very meaningful for good death.” (A8, female, 37 y.o) “When patients enter the terminal stage, some other treatments are meaningless and try not to disturb them, so that patients can quietly go through the final stage of life.” (A7, female, 39 y.o).

#### Palliative care

During the interview, most interviewees expressed that they should try their best to meet the reasonable requirements of patients, reduce invasive operations on patients, listen to the voice of patients and their families more, improve the comfort level of patients, and give more tolerance and understanding to patients and their families. “During the epidemic, patients and their families are required to reduce going out and order meals in the department. However, patients with advanced stage do have a poor appetite. Some of their families will prepare meals and send them to the first floor of the hospital building. (A9, female, 32 y.o) “When I perform a blood gas analysis or an infusion on this type of patient, if I feel that I cannot successfully puncture the vein on the first try, I will seek assistance from other colleagues to avoid subjecting the patient to the pain of a second puncture.” (A1, female, 33 y.o).

## Discussion

The aim of this study was to explore the perceptions of oncology nurses regarding end-of-life care for patients with advanced-stage cancer in China. The interviews conducted in this study revealed that oncology nurses have generated numerous ideas and understandings about end-of-life care for patients. This demonstrates their strong concern for end-of-life care issues and their utmost efforts to help patients achieve a good death. The cognition of healthcare professionals regarding a good death is influenced by traditional cultural factors, and their ability to assist patients in achieving a good death is also limited by their level of knowledge and skills.

In contemporary times, the majority of individuals pass away within the confines of a hospital setting, necessitating the presence of nurses during their final days. As patients approach the end of their lives, nurses provide companionship and support throughout this significant transition [31]. The attitudes exhibited by nurses play a pivotal role in shaping the quality of end-of-life care. A positive attitude towards death can signify that nurses possess a more effective adaptation to the practices related to end-of-life care. This also implies that they are better equipped to provide compassionate and supportive care to patients during this sensitive stage [32, 33]. By fostering positive attitudes towards the dying, nurses can overcome their own fears of death. This allows them to create a safe and supportive environment where patients can experience a peaceful and dignified process of dying. Such an atmosphere fosters a sense of respect and enables patients to feel valued as individuals during this vulnerable time [34]. The research conducted by Ceyhan et al. revealed a positive correlation between the perception of a good death and the attitudes of intensive care nurses towards providing care for patients in their final moments. These nurses exhibited favorable attitudes towards end-of-life care and possessed a strong belief in the concept of a good death. The study suggests that nurses in the intensive care setting are more inclined to embrace and prioritize the well-being and comfort of dying patients [35].

### The correct understanding and view of “good death” is the premise to realize “good death”

This is similar to the findings of Hilal Türkben Polat and others [34]. The concept of death often evokes negative emotions in patients, patient relatives, and nurses. Consequently, it is typically avoided and sometimes even considered a taboo in certain regions. Patients in Eastern countries encounter unique challenges when it comes to preparing for death. This is primarily due to lower frequencies of receiving bad news, such as diagnoses and prognoses, as well as cultural practices that discourage discussions about death. Moreover, stronger taboos surrounding death discussions exist in Eastern countries compared to Western countries [36]. In western countries, the disclosure of diagnoses is regarded as a fundamental patient right and an essential practice. Within the western ethical tradition, there is significant emphasis on providing patients with truthful information. Medical practitioners have a responsibility to respond to patients’ questions regarding their diagnosis in an honest and forthright manner [37].

Under the influence of Eastern philosophy, the challenges related to diagnosis disclosure are further magnified in Eastern countries. Traditional Eastern philosophical beliefs, such as the emphasis on harmony, collective well-being, and the idea of protecting patients from distress, can create barriers to open and direct communication about diagnoses. Balancing the values of truthfulness and preserving emotional well-being becomes a particular challenge within the Eastern cultural context [38]. In China, it is common for family members to withhold cancer diagnoses from the patient, as they believe it may help protect the patient from potential emotional distress and depression. This practice stems from a desire to shield loved ones from the potentially negative impact of such news. However, it is important to note that this approach may differ from the Western emphasis on patient autonomy and the right to access complete information about one’s own health condition [26]. As highlighted by Jiang Yu et al., the decision to withhold cancer diagnoses in China is often a collective consensus among family members. This collective decision-making process is influenced by cultural norms, where the family plays a central role in matters of health and well-being. In such cases, the family members believe that keeping the diagnosis concealed is in the best interest of the patient, aiming to maintain emotional well-being and alleviate potential distress. It is important to recognize and respect these cultural differences and the role of familial decision-making in the context of healthcare practices [39].

The concept of “Avoid death” in our traditional culture will affect the expression of the needs of terminal cancer patients. Compared with patients, their families have more difficulty accepting the concept of good death, which they believe means giving up treatment, waiting for death, and being difficult to accept psychologically [40]. The medical personnel should strengthen the family members’ correct understanding of good death and make them realize the importance of respecting the patient’s right to know and independent decision-making to realize good death, it is suggested that family decision-making should be gradually changed into a way of discussion between patients and their families, to lighten the psychological burden of both sides and let patients realize their wishes. Fully pay attention to the needs of end-stage patients and their families, and take targeted measures to help patients to achieve good death [41].

### The good death ability of nursing staff needs to be improved

The attitude of professional nurses to death greatly influences the treatment decision of terminal cancer patients and affects the quality of patients’ death. The more skilled the nursing skills, the better the communication skills, and the terminal attitude of terminal cancer patients, the better the quality of life of the patients.

Based on the interview results, it is evident that nurses have insufficient competency in implementing end-of-life care. They make efforts to help patients manage clinical symptoms and enhance the caregiving abilities of family members through their own capabilities, aiming to assist patients in a better end-of-life experience. However, the nurses’ level of competence directly affects the patient’s experience of end-of-life care. They have limited opportunities for formal end-of-life care training and education, resulting in a relative lack of knowledge in this area. The end-of-life experience is unique and personal for each individual, with most people desiring to avoid pain during this period, while others may prioritize prolonging life at any cost. End-of-life care may be provided by doctors, physicians, nurses, emergency personnel, or volunteers. However, nurses play a significant role and bear primary responsibilities in this regard [14]. A study indicated that nurses, as moral agents, possess a profound commitment to upholding the moral integrity of end-of-life care, particularly when it involves assisted death. This suggests that nurses play a crucial role in ensuring that ethical principles and values are upheld throughout the process. Their dedication to promoting the well-being and dignity of patients in these complex situations highlights their ethical and moral responsibility in providing compassionate and supportive end-of-life care [15]. In addition, another research study highlighted the indispensable role of nurses in providing compassionate care to patients in their final stages of life [16]. Nurses are entrusted with the responsibility to deliver exceptional care to terminally ill patients and their families. Insufficient knowledge has been identified as a major obstacle in providing optimal care for individuals nearing the end of their lives [18]. A lack of education and training in end-of-life care has been recognized as a significant contributing factor to insufficient recognition and management of symptoms, as well as challenges in effective communication with patients and their families [17].

At present, the level of knowledge and skills of our palliative care is not high, and they lack the skills of psychological, social, and spiritual support and are difficult to implement skillfully [42]. The limited awareness of hospice care in Mainland China can be attributed to various factors, such as the absence of systematic policy support, limited public educational campaigns, and the lack of comprehensive academic and practical curricula and training programs on hospice care. These factors have collectively contributed to the insufficient understanding and recognition of hospice care among the general public and healthcare professionals in Mainland China [43].

Healthcare professionals need professional knowledge and skills should use a variety of ways to educate professionals, and guide them not only care about patient survival rate, and quality of life, at the same time, we should also pay attention to the physical and psychological needs of incurable patients [44], educate patients with end-stage cancer and their families, provide a suitable environment and the necessary help, improve the quality of patient’s death, and meeting the needs of patients who are nearing the end of life. By enhancing the medical curriculum to include comprehensive education on hospice care and establishing hospice care programs within hospitals, opportunities can be increased for physicians, nurses, patients, and their family members to enhance their awareness and utilization of hospice care services. This would ultimately contribute to improving end-of-life care and ensuring that individuals receive the support and comfort they need during this crucial time [45]. In addition, the whole society should widely carry out life education and Death Education, guide people to look at life and death correctly, a planned, leisurely life with, a good start, and a good finish.

### Paying attention to and meeting the needs and wishes of individuals and families is the guarantee of achieving “good death”

In the Chinese cultural context, there is a strong emphasis on the centrality of the family and social relationships [12]. Family dynamics are considered crucial for a good death, and Asian populations, influenced by Confucian teachings, place great importance on the cohesion of the family and the significance of familial relationships [46]. Nurses take care of terminal cancer patients for a long time, and they are familiar with the patients and their families. Clinical nurses should play an active role as a good communication bridge, which can help them communicate their needs or promote communication among themselves, at the same time, teach family members to play a better role in family support to meet the needs of patients with end-stage cancer to receive family warmth and care [47–49].

### Limitation

This study still has some limitations. Primarily, the participants were confined exclusively to a solitary tertiary hospital, thereby potentially limiting the generalizability of the findings. To augment representativeness, future investigations could contemplate sampling participants from nontertiary hospitals. Furthermore, the inclusion of solely female nurses in the analysis neglects male nurses, thus introducing a predisposed bias into the results. Within the targeted population of this inquiry, the dearth of male nurses serving escalates the complexity of ameliorating this bias. Secondly, the study lacked the amalgamation of quantitative research, impeding the determination of specific domains and the magnitude of improvement required in nurses’ competencies. To rectify this, future research endeavors should endeavor to broaden the sample size and scope, employing quantitative research methods to scrutinize the precise cognitive facets and knowledge modules necessitating enhancement in nurses. Additionally, there exists a demand for further exploration of culturally tailored competency models in the Chinese context. This would assist in confronting and resolving the challenges impeding the current competency development process. Furthermore, interviews were conducted in Chinese and subsequently analyzed and translated into English. Despite efforts by professional English editors to guarantee accurate translation, there remains a small risk that the translation process may have influenced the study outcomes. Lastly, further exploration is still required to ascertain the appropriate cultural backdrop of our model, and to refine and address the prevailing complications encountered in the euthanasia procedure.

## Conclusion

This study explored the general cognition of nurses in the oncology department about good death from the perspective of Chinese nurses. The results showed that nurses in the oncology department had a low level of knowledge about good death, and had a correct understanding and view of “good death”. Indeed, strengthening hospice education is crucial to improve public awareness and acceptance of hospice care, leading to better quality end-of-life care. To enhance public education on hospice care, it is essential to develop and implement culturally appropriate educational programs systematically. By tailoring these programs to the specific cultural context, we can effectively address the barriers and taboos surrounding discussions about death and promote understanding and acceptance of hospice care [50]. It is the premise of realizing “good death”, and the ability of nurses should be improved. It is the guarantee of realizing “good death” to pay attention to and satisfy the needs and wishes of individuals and families.

### Electronic supplementary material

Below is the link to the electronic supplementary material.

Supplementary Material 1

## Data Availability

The datasets generated during the current study are available from the corresponding author on reasonable request.

## References

[1] Sung H, Ferlay J, Siegel RL, Laversanne M, Soerjomataram I, Jemal A, Bray F (2021). Global Cancer statistics 2020: GLOBOCAN estimates of incidence and Mortality Worldwide for 36 cancers in 185 countries. CA Cancer J Clin.

[2] Wei W, Zeng H, Zheng R, Zhang S, An L, Chen R, Wang S, Sun K, Matsuda T, Bray F, He J (2020). Cancer registration in China and its role in cancer prevention and control. Lancet Oncol.

[3] Feng RM, Zong YN, Cao SM, Xu RH (2019). Current cancer situation in China: good or bad news from the 2018 Global Cancer statistics?. Cancer Commun (Lond).

[4] Roger C, Morel J, Molinari N, Orban JC, Jung B, Futier E, Desebbe O, Friggeri A, Silva S, Bouzat P, Ragonnet B, Allaouchiche B, Constantin JM, Ichai C, Jaber S, Leone M, Lefrant JY, Rimmelé T (2015). Practices of end-of-life decisions in 66 southern French ICUs 4 years after an official legal framework: a 1-day audit. Anaesth Crit Care Pain Med.

[5] Kawasaki Y (2014). Consultation techniques using shared decision making for patients with cancer and their families. Clin J Oncol Nurs.

[6] Ho AH, Leung PP, Tse DM, Pang SM, Chochinov HM, Neimeyer RA, Chan CL (2013). Dignity amidst liminality: healing within suffering among Chinese terminal cancer patients. Death Stud.

[7] Joarder T, Cooper A, Zaman S (2014). Meaning of death: an exploration of perception of elderly in a Bangladeshi village. J Cross Cult Gerontol.

[8] Kumar P, Wright AA, Hatfield LA, Temel JS, Keating NL (2017). Family perspectives on Hospice Care experiences of patients with Cancer. J Clin Oncol.

[9] Huang YT, Wang YW, Chi CW, Hu WY, Lin R, Shiao CC, Tang WR (2020). Differences in medical costs for end-of-life patients receiving traditional care and those receiving hospice care: a retrospective study. PLoS ONE.

[10] Shen VW, Yang C, Lai LL, Chen YJ, Huang HH, Tsai SH, Hsu TF, Yen DH. (2021) Emergency Department Referral for Hospice and Palliative Care Differs among patients with different end-of-life trajectories: a retrospective cohort study. Int J Environ Res Public Health 18. 10.3390/ijerph18126286 PMC8296068 34200689

[11] Cheng Q, Duan Y, Zheng H, Xu X, Khan K, Xie J, Chen Y (2021). Knowledge, attitudes and preferences of palliative and end-of-life care among patients with cancer in mainland China: a cross-sectional study. BMJ Open.

[12] Krikorian A, Maldonado C, Pastrana T (2020). Patient’s perspectives on the notion of a good death: a systematic review of the literature. J Pain Symptom Manage.

[13] Üzen Cura Ş (2021). Nursing students’ spiritual orientations and their attitudes toward the principles of dying with dignity: a sample from Turkey. J Relig Health.

[14] Noome M, Dijkstra BM, van Leeuwen E, Vloet LCM (2017). Effectiveness of supporting intensive care units on implementing the guideline ‘End-of-life care in the intensive care unit, nursing care’: a cluster randomized controlled trial. J Adv Nurs.

[15] Ghezelsefli Z, Ahmadi F, Mohammadi E (2020). End-of-Life Care provided for Cancer patients: a Metasynthesis Study. Holist Nurs Pract.

[16] Mahan P, Taggart H, Knofczynski G, Warnock S (2019). Transforming nursing students’ attitudes toward End-of-Life Care. J Hosp Palliat Nurs.

[17] McIlfatrick S, Connolly M, Collins R, Murphy T, Johnston B, Larkin P (2017). Evaluating a dignity care intervention for palliative care in the community setting: community nurses’ perspectives. J Clin Nurs.

[18] Aslakson RA, Wyskiel R, Thornton I, Copley C, Shaffer D, Zyra M, Nelson J, Pronovost PJ (2012). Nurse-perceived barriers to effective communication regarding prognosis and optimal end-of-life care for surgical ICU patients: a qualitative exploration. J Palliat Med.

[19] Colaizzi PF. Psychological research as the phenomenologist views it. In: Valle RS, King M, editors. Existential-phenomenological Alternatives for psychology. Oxford University Press; 1978. p. 6.

[20] Tong A, Sainsbury P, Craig J (2007). Consolidated criteria for reporting qualitative research (COREQ): a 32-item checklist for interviews and focus groups. Int J Qual Health Care.

[21] Gurdogan EP, Aksoy B, Kinici E (2022). The Concept of a good death from the perspectives of Family caregivers of Advanced Cancer patients. Omega (Westport).

[22] Lyerly HK, Fawzy MR, Aziz Z, Nair R, Pramesh CS, Parmar V, Parikh PM, Jamal R, Irumnaz A, Ren J, Stockler MR, Abernethy AP (2015). Regional variation in identified cancer care needs of early-career oncologists in China, India, and Pakistan. Oncologist.

[23] Billings JA, Block S (1997). Palliative care in undergraduate medical education. Status report and future directions. JAMA.

[24] Hahne J, Liang T, Khoshnood K, Wang X, Li X (2020). Breaking bad news about cancer in China: concerns and conflicts faced by doctors deciding whether to inform patients. Patient Educ Couns.

[25] Ling DL, Yu HJ, Guo HL (2019). Truth-telling, decision-making, and ethics among cancer patients in nursing practice in China. Nurs Ethics.

[26] Liu Y, Yang J, Huo D, Fan H, Gao Y (2018). Disclosure of cancer diagnosis in China: the incidence, patients’ situation, and different preferences between patients and their family members and related influence factors. Cancer Manag Res.

[27] Rao A, Ekstrand M, Heylen E, Raju G, Shet A (2016). Breaking Bad News: patient preferences and the role of Family members when delivering a Cancer diagnosis. Asian Pac J Cancer Prev.

[28] Griggs C (2010). Community nurses’ perceptions of a good death: a qualitative exploratory study. Int J Palliat Nurs.

[29] Yamagishi A, Morita T, Miyashita M, Igarashi A, Akiyama M, Akizuki N, Shirahige Y, Eguchi K (2012). Pain intensity, quality of life, quality of palliative care, and satisfaction in outpatients with metastatic or recurrent cancer: a Japanese, nationwide, region-based, multicenter survey. J Pain Symptom Manage.

[30] Miyashita M, Morita T, Sato K, Hirai K, Shima Y, Uchitomi Y (2008). Factors contributing to evaluation of a good death from the bereaved family member’s perspective. Psychooncology.

[31] Loerzel VW, Conner N (2016). Advances and challenges: student reflections from an online death and dying course. Am J Hosp Palliat Care.

[32] Gillan PC, van der Riet PJ, Jeong S (2014). End of life care education, past and present: a review of the literature. Nurse Educ Today.

[33] Henoch I, Browall M, Melin-Johansson C, Danielson E, Udo C, Johansson Sundler A, Björk M, Ek K, Hammarlund K, Bergh I, Strang S (2014). The Swedish version of the Frommelt attitude toward care of the dying scale: aspects of validity and factors influencing nurses’ and nursing students’ attitudes. Cancer Nurs.

[34] Türkben Polat H. Nurses’ Perceptions on Good Death and Their Attitudes Towards the Care of Dying Individuals. Omega (Westport; 2022. p. 302228221100638. 10.1177/00302228221100638 35544677

[35] Ceyhan Ö, Özen B, Zincir H, Şimşek N, Başaran M (2018). How intensive care nurses perceive good death. Death Stud.

[36] Xiao J, Ding J, Huang C (2022). Notion of a good death for patients with cancer: a qualitative systematic review protocol. BMJ Open.

[37] Zolkefli Y (2018). The Ethics of Truth-Telling in Health-Care settings. Malays J Med Sci.

[38] Beyraghi N, Mottaghipour Y, Mehraban A, Eslamian E, Esfahani F (2011). Disclosure of Cancer Information in Iran: a perspective of patients, Family Members, and Health professionals. Iran J Cancer Prev.

[39] Jiang Y, Liu C, Li JY, Huang MJ, Yao WX, Zhang R, Yao B, Du XB, Chen J, Xie K, Zhao X, Wei YQ (2007). Different attitudes of Chinese patients and their families toward truth telling of different stages of cancer. Psychooncology.

[40] Emanuel EJ, Emanuel LL (1998). The promise of a good death. Lancet.

[41] Cipolletta S, Oprandi N (2014). What is a good death? Health care professionals’ narrations on end-of-life care. Death Stud.

[42] van Laarhoven HW, Schilderman J, Verhagen CA, Vissers KC, Prins J (2011). Perspectives on death and an afterlife in relation to quality of life, depression, and hopelessness in cancer patients without evidence of disease and advanced cancer patients. J Pain Symptom Manage.

[43] Ni K, Gong Y, Li F, Cao X, Zhang H, Chu H, Li T, Mairipaiti A, Zhao Y, Li N (2019). Knowledge and attitudes regarding hospice care among outpatients and family members in two hospitals in China. Med (Baltim).

[44] Brumley R, Enguidanos S, Jamison P, Seitz R, Morgenstern N, Saito S, McIlwane J, Hillary K, Gonzalez J (2007). Increased satisfaction with care and lower costs: results of a randomized trial of in-home palliative care. J Am Geriatr Soc.

[45] Yin Z, Li J, Ma K, Ning X, Chen H, Fu H, Zhang H, Wang C, Bruera E, Hui D (2017). Development of Palliative Care in China: a tale of Three cities. Oncologist.

[46] Li J (2001). Chinese conceptualization of learning. Ethos.

[47] Walczak A, Butow PN, Tattersall MH, Davidson PM, Young J, Epstein RM, Costa DS, Clayton JM (2017). Encouraging early discussion of life expectancy and end-of-life care: a randomised controlled trial of a nurse-led communication support program for patients and caregivers. Int J Nurs Stud.

[48] Hattori K, McCubbin MA, Ishida DN (2006). Concept analysis of good death in the Japanese community. J Nurs Scholarsh.

[49] Becker CL, Arnold RM, Park SY, Rosenzweig M, Smith TJ, White DB, Smith KJ, Schenker Y (2017). A cluster randomized trial of a primary palliative care intervention (CONNECT) for patients with advanced cancer: protocol and key design considerations. Contemp Clin Trials.

[50] Lin H, Ko E, Wu B, Ni P. (2022) Hospice Care preferences and its Associated factors among Community-Dwelling residents in China. Int J Environ Res Public Health 19. 10.3390/ijerph19159197 PMC9368034 35954548

